# Real-World Data Analysis of CDK4/6 Inhibitor Therapy—A Patient-Centric Single Center Study

**DOI:** 10.3390/cancers16091760

**Published:** 2024-05-01

**Authors:** Isabell Ge, Kai Berner, Marlene Mathis, Catherine Hensgen, Sebastian Mayer, Thalia Erbes, Ingolf Juhasz-Böss, Jasmin Asberger

**Affiliations:** 1 Department of Gynecology and Obstetrics, University Hospital Basel, 4031 Basel, Switzerland; isabellxiang.ge@usb.ch; 2 Breast Center, University Hospital Basel, University of Basel, 4001 Basel, Switzerland; 3 Department of Obstetrics and Gynecology, Medical Center-University of Freiburg, 79106 Freiburg, Germany; 4 Faculty of Medicine, University of Freiburg, 79106 Freiburg, Germany; 5 Department of Gyneaecology and Obstetrics, Diako Mannheim, 68163 Mannheim, Germany; 6 Department of Gynaecology and Obstetrics, Hospital Krumbach, 86381 Krumbach, Germany

**Keywords:** breast cancer, CDK4/6 inhibitors, progression-free survival (PFS), time-to-treatment failure (TTF), progesterone receptor (PR), therapeutic benefit, prediction, multivariate analysis, metastasis, adverse events, personalized treatment

## Abstract

**Simple Summary:**

In this study, we investigated the effectiveness of the targeted cancer medication CDK4/6 inhibitors, in patients with breast cancer. These medicaments have been used for nearly a decade, but how well they work can vary greatly from one patient to another. By examining the experiences of 86 patients over a period from November 2016 to May 2020, we aimed to understand what factors might predict better or worse outcomes for patients. We discovered that certain characteristics, like the level of the progesterone receptor in the tumor and whether the cancer had spread to multiple locations or the liver, played a significant role in how long patients benefited from treatment without progression. The study also found that management characteristics during treatment could significantly affect patient outcomes. These insights are crucial for developing personalized management strategies that could lead to better outcomes for people with breast cancer.

**Abstract:**

Background: The quest to comprehend the real-world efficacy of CDK4/6 inhibitors (CDKis) in breast cancer continues, as patient responses vary significantly. Methods: This single-center retrospective study evaluated CDKi use outside the trial condition from November 2016 to May 2020. Progression-free survival (PFS), time-to-treatment failure (TTF), short-term and prolonged treatment benefit (≥4 and ≥10 months), as well as prognostic and predictive markers were assessed with Kaplan–Meier and multivariate regression analyses. Results: Out of 86 identified patients, 58 (67.4%) had treatment failure of which 40 (46.5%) were due to progression. Median PFS and TTF were 12 and 8.5 months, respectively. A total of 57 (66.3%) and 42 (48.8%) patients experienced short-term and prolonged treatment benefit. Independent, significant predictors for PFS were progesterone receptor expression (HR: 0.88), multiple metastatic sites (HR: 2.56), and hepatic metastasis (HR: 2.01). Significant predictors for TTF were PR expression (HR: 0.86), multiple sites (HR: 3.29), adverse events (HR: 2.35), and diabetes (HR: 2.88). Aside from tumor biology and adverse events, treatment modifications like pausing and switching of CDKi were predictive for short-term (OR: 6.73) and prolonged (OR: 14.27) therapeutic benefit, respectively. Conclusions: These findings emphasize the importance of tailored treatment strategies, highlighting the role of PR expression, metastatic burden, and therapeutic adjustments in optimizing patient outcomes in real-world breast cancer management.

## 1. Introduction

Breast cancer (BC) is the most common type of cancer in women worldwide and accounted for 684,996 BC-associated deaths in 2020 [1]. Based on the histologic, molecular biology, and ultimately genomic characteristics, BC is divided into several subtypes with individual corresponding treatment approaches and prognosis [2,3]. Ductal, lobular, mucinous (colloid), tubular, medullary and papillary carcinomas [4,5,6,7], as well as luminal A, luminal B, HER2-enriched and triple negative can be distinguished according to the underlying gene expression profile [2,4,8,9,10,11,12]. In terms of therapeutic management, a combination of surgery, radiotherapy, endocrine therapy (ET), chemotherapy or varying targeted therapies are common [2,13]. However, individual disease management depends on a number of disease characteristics, e.g., TNM status and molecular biology, wherein both increase the treatment complexity [2,13].

Since their introduction in 2015, CDK4/6 inhibitors (CDKis) have become a common systemic therapy in BC. They target the cyclin-dependent kinases CDK 4 and 6, which themselves are key drivers of abnormal cellular proliferation in BC [14,15,16]. Approved first-generation CDKis are Palbociclib, Ribociclib, and Abemaciclib. In 2015, Palbociclib obtained approval as the first CDKi based on the results of the PALOMA-1/TRIO-18 trial. Within this study, the addition of Palbociclib to Letrozole in patients with advanced, ER-positive and HER2-negative BC demonstrated a prolongation of PFS of ten months (20.2 months vs. 10.2 months, hazard ratio (HR) 0.49, *p* = 0.0004) [17]. Subsequently, PALOMA-2 verified these results [18] and PALOMA-3 showed comparable outcomes for the combination with Fulvestrant [19]. The MONALEESA-2 trial and the MONARCH-3 trial presented similar results for Ribociclib [20] and Abemaciclib [21], respectively. Recently, for high-risk BC patients in the adjuvant setting, the MONARCH-E and NATALEE trial have also demonstrated a significant prognostic benefit by adding Abemaciclib and Ribociclib to endocrine maintenance therapy [22,23]. Still, there also appears to be a BC patient subgroup which discontinues CDK4/6 therapy at an early stage due to insufficient, missing response to therapy [24].

Outside clinics trials, CDKi therapy shows varied success. This has called for clinical markers for a more personalized approach to CDKi therapy early on. Still, after almost ten years, there is only limited data regarding this issue. Subgroup analyses of the big approval studies have extensively analyzed patient parameters targeting this question, with no significant disadvantage of any subgroup [25]. Pooling the data of the MONARCH-2 and -3 trials, Di Leo et al. identified different clinical parameters that predict therapy responsiveness to Abemaciclib in 2018. Bone-only disease, liver metastases, tumor grade, progesterone receptor (PR) status, and ECOG performance status emerged to have potential value [26]. In a more extensive study, Piezzo et al. analyzed 2802 patients from the eight aforementioned randomized controlled trials without any subgroup specific survival benefits [24]. Additionally, a treatment-free interval of three years or more has also been suggested as a clinical marker for therapy success of Abemaciclib [15]. After the first years of usage experience, powerful real-world data are available to address this unanswered question. Two larger trials have been conducted. The PRAEGNANT trial verified the results from clinical RCTs by showing a median PFS of 24.7 months [27], while the POLARIS trial exceeded the value with a PFS of 32.2 months [28]. However, therapy discontinuation due to other reasons than progression as well as predictive parameters in terms of therapy response were not assessed [27].

The approach of the present real-world evidence (RWE) study extends beyond the conventional endpoint of progression-free survival (PFS) and delves into the role of analyzing time-to-treatment failure (TTF), a comprehensive metric encompassing disease progression, treatment discontinuation, and overall treatment durability [29]. Additionally, we aim to identify factors influencing PFS, TTF, and treatment benefit in metastatic BC patients receiving CDKi, a study question which has been rarely explored. Such insights are pivotal for clinicians, researchers, and policymakers alike, as they contribute to informed decision-making, patient-centered care, and the continuous refinement of treatment strategies in the evolving landscape of BC management.

## 2. Materials and Methods

### 2.1. Study Design and Patient Selection

**Figure 1:**
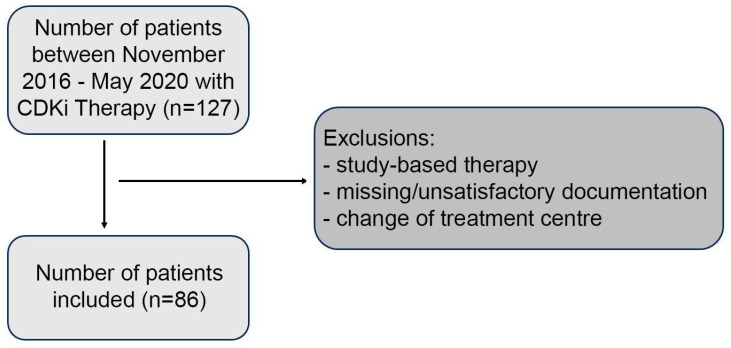
Flow chart of the exclusion criteria of the study population.

This investigation is a retrospective study, encompassing patients receiving CDKi therapy at the Department of Gynecology and Obstetrics, University Clinic Freiburg, Germany, from November 2016 to May 2020. Eligible patients were those treated with Palbociclib, Ribociclib, or Abemaciclib. Comprehensive data regarding tumor and patient characteristics were extracted from electronic patient records. Exclusion criteria were devised to ensure the collection of authentic real-world data, excluding patients participating in study-based therapy regimens (e.g., MONARCH-E), those with insufficient clinical documentation, unknown clinical outcomes, or those who transferred to another treatment center during their therapy course. Figure 1 provides a detailed overview of the patient exclusion process. Upon thorough examination of all patient records, a specific set of variables was identified and analyzed. Subsequent refinement, involving the removal of variables with excessive missing data, unclear relevance or redundancy led to a distilled dataset for both univariate and multivariate analysis (the extracted characteristics are detailed in Supplemental Materials File S1).

### 2.2. Outcome Measures

To capture the breadth of therapeutic outcomes, from clinical efficacy to tangible benefits on patient well-being, the following four endpoints were chosen for exploration:

1. PFS, which was defined as the time from beginning of the therapy until progression of disease.
2. TTF, defined as the time from beginning of the therapy to discontinuation of the treatment for any reason, including progression of disease and treatment toxicity.
3. Short-term treatment benefit: treatment period of 4 months or longer without discontinuation of any reason.
4. Prolonged treatment benefit: treatment period of 10 months or longer without discontinuation of any reason.

### 2.3. Statistical Analysis

Statistical analyses were performed using SAS OnDemand (SAS Campus Drive, Cary, NC, USA). Descriptive analyses summarizing baseline patients and tumor characteristics are presented as n (%), mean and/or median (minimum-maximum range), as applicable. 

Kaplan–Meier analysis was used to evaluate median PFS and median TTF. Multivariate cox proportional hazard regression was performed to identify independent prognostic markers for PFS and TTF. Multivariate logistic regression was used to identify independent predictive markers for treatment benefit beyond 4 and 10 months of CDKi therapy. For multivariate analyses, missing values were imputed with mean/median values. Using the stepwise selection method, only significant predictor variables to the four endpoints above were included in the multivariate regression models.

## 3. Results

### 3.1. Descriptive Analysis

**Figure 2:**
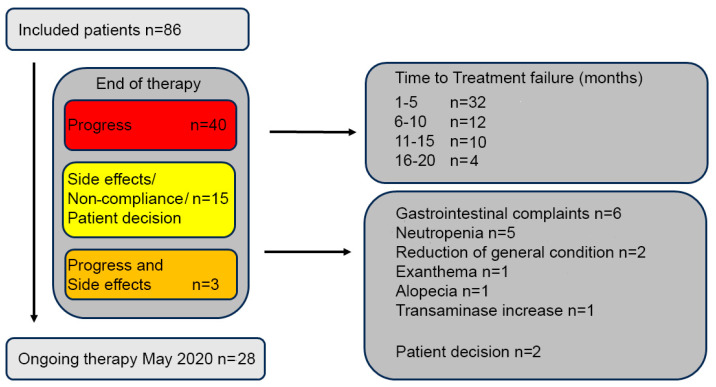
Flow chart of the cohort and reasons of treatment failure.

In this single-center RWE study conducted from November 2016 to May 2020, 86 patients were eligible for evaluation. A total of 58 patients (58/86, 67.4%) discontinued their treatment during the observational period, most frequently due to disease progression in 40 patients (40/86, 46.5%). Discontinuation of treatment due to adverse events (AE) or upon the patient’s request occurred in 15 patients (15/86, 17.4%), while 3 patients (3/86, 3.5%) discontinued due to both disease progression and AE. At the time of data analysis, 28 patients (28/86, 32.6%) were still receiving treatment. Figure 2 displays the reasons for treatment failure in the analyzed patient cohort. Median PFS was 12 months, spanning from 2 to 20 months. The median TTF was 8.5 months, ranging from 1 to 20 months. A total of 57 patients (57/86, 66.3%) experienced a therapeutic benefit of at least 4 months, while a prolonged benefit, lasting more than 10 months, was seen in 42 patients (42/86, 48.8%).

#### 3.1.1. Descriptive Analysis of the Patient Cohort

Characteristics of the patient cohort and descriptive analyses are listed in Table 1 and Table 2. At the initial diagnosis, the average age was 56.3 years (range: 28 to 86 years), 62.4 years at metastasis, and 64.3 years at the start of CDKi. The median period from metastatic disease diagnosis to CDKi initiation was 5 months (range: 0 to 155 months). Moreover, 45.4% received CDKi as the first-line treatment. A substantial majority, 86.1% (74/86), were treated with Palbociclib, followed by Ribociclib in 15.1% (13/86), and Abemaciclib in 5.8% of patients (5/86). Furthermore, five patients (5/86, 5.8%) underwent a CDKi switch.

#### 3.1.2. Tumor Biology Characteristics

Tumor biology analysis showed a median Ki67 of 30% (range: 3 to 80%), as well as median ER positivity of 95% (range: 0 to 100%) and PR positivity of 62.5% (range: 0 to 95%) at any time of the disease. Tumor grading identified 1.2% (1/86) as Grade 1, 65.1% (56/86) as Grade 2, and 31.4% (27/86) as Grade 3. 

#### 3.1.3. Patient Characteristics

Patient demographics revealed a mean BMI of 27.2 (range: 17.2 to 49.1). Moreover, 79.1% (68/86) were postmenopausal. Positive family history was noted in 27.9% (24/86), relevant comorbidities were present in 65.1% (56/86), including diabetes mellitus in 12.8% (11/86).

#### 3.1.4. Tumor Stage at Diagnosis and Treatment History

Prior to the diagnosis of metastases, 57 patients (57/86, 66.3%) had an earlier diagnosis of primary BC (secondary metastases). The remaining patients were primarily metastatic (29/86, 33.7%). In 65.1% (56/86) of cases, the metastases had spread to multiple sites, 60.5% (52/86) having visceral metastases. In 30 cases (30/86, 34.9%), metastases were limited to a single site, of which 21 cases were bone-only (21/86, 24.4%).

#### 3.1.5. Prior Treatments

Prior to enrollment either at primary diagnosis or in metastasis, 70.9% (61/86) underwent surgical treatment, with mastectomy in 38.4% (33/86) and axillary dissection in 45.4 (39.0%). Radiation therapy and chemotherapy were administered in 82.6% (71/86) and 68.6% (59/86), respectively. Neoadjuvant chemotherapy at initial diagnosis was given to 17 patients (17/86, 19.9%). Out of the 57 patients with primary BC, previous adjuvant hormonal therapy was given in 47 patients (47/57, 82.5%). Resistance to endocrine therapy developed in 28 out of the 47 patients (28/47, 59.6%).

### 3.2. Multivariate Proportional Hazards and Logistic Regression

Variables significantly associated with PFS, TTF, and therapeutic benefit at four and ten months identified by multivariate regression are displayed in Table 3. Detailed univariate regression results for tumor biology (estrogen receptor (ER) and Ki67 expression), location of metastatic sites, and previous treatments are displayed in Supplementary Materials Table S1.

#### 3.2.1. Progression-Free Survival (PFS)

**Figure 3:**
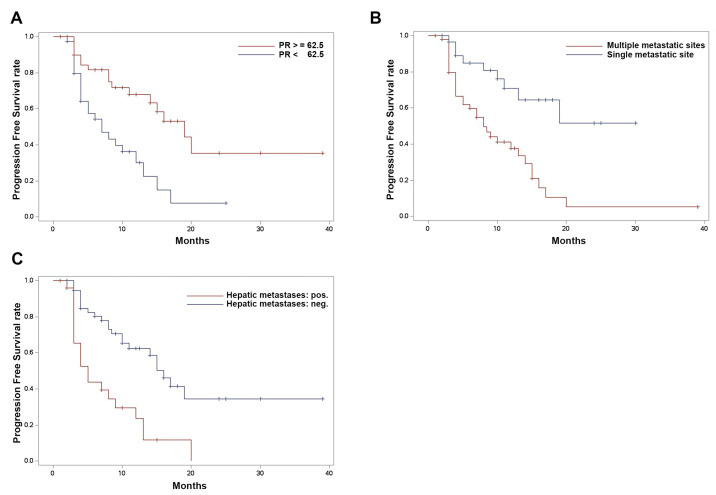
Kaplan–Meier analysis of progression-free survival comparing (**A**) progesterone receptor status over vs. under the median (62.5%); *p* = 0.0021 by log-rank. (**B**) Multiple metastatic sites vs. single metastatic site; *p* = 0.0007 by log-rank. (**C**) Presence vs. absence of hepatic metastases; *p* = 0.0004 by log-rank.

Multivariate analysis identified three independent variables significantly associated with PFS. A high PR status at primary diagnosis or in metastasis was associated with longer PFS, evidenced by an HR of 0.880 (95% confidence interval (CI): 0.804–0.900, *p*-value: 0.006) per 10% PR increase. Additionally, the presence of multiple metastatic sites and hepatic metastases significantly decreased PFS, with HRs of 2.557 (CI: 1.135–5.763, *p*-value: 0.024) and 2.009 (CI: 1.034–3.903, *p*-value: 0.040), respectively. Kaplan–Meier curves are displayed in Figure 3.

#### 3.2.2. Time-to-Treatment Failure (TTF)

**Figure 4:**
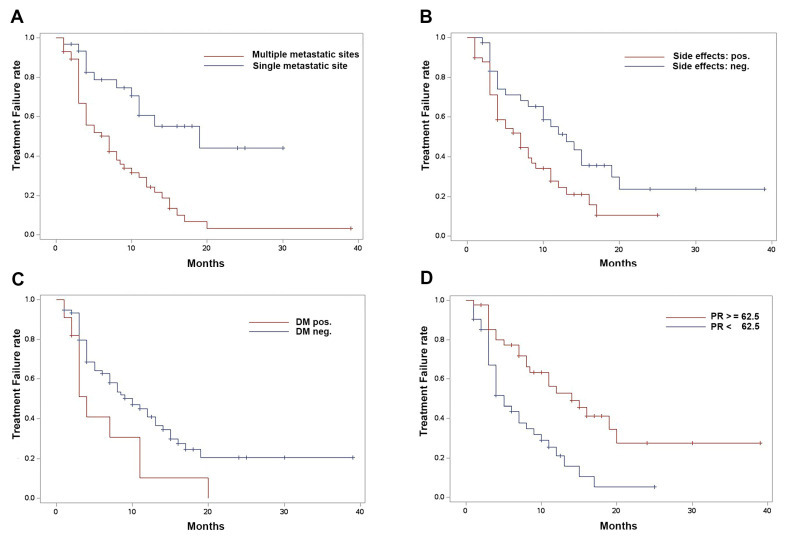
Kaplan–Meier analysis of time-to-treatment failure comparing (**A**) multiple metastatic sites vs. single metastatic site; *p* < 0.0001 by log-rank. (**B**) Presence vs. absence of adverse events; *p* = 0.0088 by log-rank. (**C**) Presence and absence of diabetes mellitus; *p* = 0.0460 by log-rank. (**D**) Progesterone receptor status over vs. under the median (62.5%); *p* = 0.0005 by log-rank.

For TTF, our analysis revealed the significance of PR expression, adverse events (AEs), multiple metastatic sites, and pre-existing diabetes mellitus as independent predictors. High PR expression was significantly associated with longer TTF (HR: 0.858, CI: 0.792–0.929, *p*-value: 0.0002 per 10% PR increase) while the occurrence of AE (HR: 2.346, CI: 1.318–4.176, *p*-value: 0.0037), the presence of multiple metastatic sites (HR: 3.290, CI: 1.699–6.369, *p*-value: 0.0004), and diabetes mellitus (HR: 2.882, CI: 1.346–6.171, *p*-value: 0.0065) were significantly associated with lower TTF. Kaplan–Meier curves are displayed in Figure 4.

#### 3.2.3. Therapeutic Benefit beyond 4 Months

High PR expression throughout the disease was positively associated with achieving therapeutic benefit at 4 months (OR: 1.220, CI: 1.047–1.423, *p*-value: 0.011 per 10% PR increase). The absence of early AE significantly increased the likelihood of benefit (OR: 4.693, CI: 1.396–15.771, *p*-value: 0.012), as did pausing ongoing CDKi therapy (OR: 6.725, CI: 1.742–25.963, *p*-value: 0.005), and a latency period between metastatic diagnosis and CDKi initiation of less than 5 months (OR: 3.485, CI: 1.150–10.564, *p*-value: 0.027).

#### 3.2.4. Therapeutic Benefit beyond 10 Months

For an extended therapeutic benefit of 10 months or more, tumor grading, the occurrence of adverse events, multiple metastases, and CDKi switching were significant predictors. High tumor grading decreased the likelihood of a longer-term benefit beyond 10 months (OR: 0.155, CI: 0.045–0.534, *p*-value: 0.003 per increase of 1 grade), as did experiencing AE in general (OR: 0.284, CI: 0.101–0.794, *p*-value: 0.017). The presence of only a single metastatic site (OR: 4.445, CI: 1.383–12.906, *p*-value: 0.0114) and undergoing a CDKi switch (OR: 14.267, CI: 1.089–186.96, *p*-value: 0.043) were significantly associated with extended therapeutic benefit.

## 4. Discussion

With the practice-changing introduction of CDKi in the treatment of BC, the therapeutic landscape has changed tremendously and is shifting even more to neoadjuvant, adjuvant, HER2-targeted, and even maintaining regimen. Despite the significant advancements in PFS demonstrated in landmark clinical randomized controlled trials (RCTs) such as PALOMA, MONALEESA, and MONARCH, the post-market routine treatment experience of patient cohorts can demonstrate a discrepant reality, resulting in a substantial gap. Pragmatic RWE studies like in the present case help bridge this gap by not only assessing the performance of medications in a realistic setting with less strict exclusion or inclusion criteria, but also by exploring patient benefit. Based on our results, we discuss PFS discrepancy between the real world and RCTs, TTF, and therapy benefit as relevant outcomes, as well as predictive characteristics for those outcome measures.

### 4.1. PFS (Progression-Free Survival)—Discrepancies between RCTs (Randomized Controlled Trials) and Real-World Conditions

In the present RWE study, the observed median PFS of 12 months under CDKi was lower than reported in pivotal registration trials: 24.8 months in PALOMA-2 for Palbociclib [18], 25.3 months in MONALEESA-2 for Ribociclib [30], and 28.2 months in MONARCH-3 for Abemaciclib [23].

When comparing the present RWE study to the three RCTs, we found several differences in the characteristics of the patient cohorts: performance status, menopausal status, prior treatments, choice of aromatase inhibitor, endocrine resistance, and pattern of metastatic spread. The differences are summarized in Table 4. 

The Eastern Cooperative Oncology Group performance status (ECOG-PS) and patient-reported outcomes on physical functioning have been demonstrated as independent predictors of PFS [31]. The prerequisite ECOG-PS was ≤1 in the MONALEESA and MONARCH studies, and ≤2 in PALOMA, whereas our study was not limited to a specific ECOG. Notably, 58% of our patients had relevant comorbidities reflecting a real-world scenario with patients who may not always be in perfect health and instead can be limited in physical and psychological functioning. Another differing factor is the menopausal status. Premenopausal BC is associated with more aggressive features and worse prognosis [32]. Compared to the RCTs, in which only postmenopausal patients were allowed, >20% of our patients were premenopausal, implying yet again a population with worse clinical outcome in general.

Additionally, 36% of our patients received previous chemotherapy for metastatic disease, contrasting with the RCT populations in which no prior chemotherapy treatment in the metastatic disease was allowed. Thus, the total rate of prior chemotherapy including adjuvant and neoadjuvant regimen was also higher in our cohort with 69% compared to 48% in PALOMA and 39% in MONARCH, indicating a more heavily pretreated population in the real-world setting. In our cohort, less than half of the patients received CDKi in the first line (45%) and over one fourth of the patients received their CDKi in the third-line or beyond (28%). In the PRAEGNANT study, it was shown that median PFS was considerably worse for patients who received CDKi in the second or third line (8.7 and 4.7 months, respectively) compared to first-line treatment (24.7 months) [27]. This is in line with the results from our study, displaying a considerably longer median PFS for first-line treatment (17 months) compared to second line (10 months) or beyond (5.5 months). This suggests that the most substantial benefit of combination therapy may be confined to the first-line setting, prompting a re-evaluation of treatment strategies in subsequent lines. Another difference between our RWE analysis and study conditions lies in the inclusion criteria related to disease-free survival (DFS) after endocrine therapy. All three RCTs required a minimum DFS of 12 months, excluding endocrine-resistant tumors, which however, accounted for 60% of our real-world population. Since CDKis are given in combination with aromatase inhibitors, previous treatment response to endocrine therapy impacts the efficacy of the combination therapy. Aside from that, the choice of endocrine therapy (aromatase inhibitor or selective estrogen receptor degrader) as a combination partner may also have an impact on treatment outcome. In the above-mentioned three RCTs, CDKi was given together with Letrozol. In the real-world and our study, both Letrozole and Fulvestrant were used. PALOMA-3 analyzed CDKi in combination with Fulvestrant in patients who had disease progression after ET [33]. Similar to our case, the median PFS was much shorter (8.9 months) compared to the other three RCTs using Letrozole in patients without endocrine resistance, again displaying the potential variability in treatment response based on the endocrine sensitivity and substance used. 

Lastly, metastatic spread and bone-only disease were distributed similarly across the three RCTs with bone-only disease in 21–23% of cases and visceral involvement in 48% and 53% for PALOMA-2 and MONARCH-2, respectively, as well as liver or lung involvement in 54% for MONALEESA-3. In this study, there were higher rates of visceral involvement with 61%. This indicates a higher proportion of patients with a generally worse prognosis and likely lower response to ET [34]. 

Beyond patient, tumor characteristics, and prior treatments, the RWD revealed significant variations in the management and course of therapy. Dose reductions were less common in the present study (23.3%) compared to trials like PALOMA, where they occurred in 36% of cases. Discontinuation due to AEs was also higher in our cohort, exceeding 20%, whereas clinical trials reported rates below 10%. These findings, especially when integrated with the results from our multivariate analyses, highlight the importance of therapeutic and a sound and patient-centric management approach. 

In sum, the discrepancies between the real world and the RCTs PALOMA, MONALEESA, and MONARCH are clearly impacting PFS in the real-world setting and must be taken into account in clinical routine when deciding on therapy options and for patient consultations. They also highlight that practitioners have to interpret results from clinical trials carefully and that personalized approaches are needed when consulting and managing patients. 

### 4.2. Time-to-Treatment Failure—An Objective and Patient-Centric Outcome Measure

As mentioned above, personalization and patient-centricity can become unnoticed and disregarded in the harsh RCT conditions, but are crucial for optimizing treatment strategies in the real world. Therefore, the consideration of TTF is particularly valuable, offering a broader understanding of treatment efficacy and durability beyond traditional metrics like PFS or OS. TTF mirrors not only disease progression, but also therapy discontinuation for any reason. Therapeutic substances can be administered beyond progression, thus prolonging TTF, but on the other hand, can be discontinued early despite displaying antineoplastic efficacy due to severe AEs, non-compliance or on patient request, thus shortening TTF [35]. This variation underscores the importance to display the realistic therapeutic landscape to ease understanding of the full spectrum of patient experiences and outcomes. However, even in the real world, the analysis of TTF has been scarce [36,37,38]. 

In our cohort, we observed a median TTF of 8.5 months with 17.4% (15/86) of patients discontinuing their treatment due to other reasons than disease progression. Similar results were reported by another RWE study reporting a median TTF of 8.3 months and 12.8% of patients discontinuing treatments due to AEs or other reasons [39]. Likewise, Collins et al. did not focus on PFS, but chose the endpoint median time-to-first subsequent treatment or death which was only 10 months instead of >20 months in the RCTs [40]. This discrepancy paints a more realistic picture about the course of therapy and what providers or patients have to be prepared for when using CDKi. In the end, it is not only the disease progression defined by imagery or tumor markers which may impact quality of life or psychological well-being, but rather the repeated discontinuation and changes in medications entailing disappointment, new side effects, uncertainty concerning efficacy, and anxiety when the next medication will be discontinued again. 

### 4.3. Independent Predictors for Survival and Therapeutic Benefit

Building on our observations of the discrepancies between RCT and real-world conditions, we further delved into whether independent markers predicting PFS, TTF, and treatment benefit could be identified in our patient cohort. 

In our multivariate statistical analyses, PR status emerged as an independent predictor for longer PFS, TTF, and treatment benefit beyond 4 months. Despite ER expression also displaying significant associations with PFS, TTF, and treatment benefit in the univariate analysis (Supplementary Materials Table S1), the association became insignificant in the multivariate analysis when PR was added, marking PR as a more specific predictor than ER. While Ki67 showed no association with any outcomes, grading was associated with prolonged benefit ≥ 10 months. These results are in line with exploratory analyses within the framework of the RCT MONARCH [26] and other RWE analyses [41], emphasizing the significance of tumor biology for the estimation of individual prognosis. Both high PR and low grading are indicators of the less aggressive and prognostically more favorable luminal A subtype BC; hence, explaining their predictive value for longer survival and treatment benefit [42]. The analysis also highlighted that single-site metastasis was significant for PFS, TTF, and prolonged benefit beyond 10 months. While single-site metastasis may indicate earlier detection and lower tumor burden, the presence of multiple metastatic [43] sites is associated with considerably worse survival [43]. Aside from the number of metastatic sites, hepatic involvement in our study was additionally associated with shorter PFS, an effect similarly reported in MONARCH [26]. However, therapy benefit, whether short-term or prolonged, was not associated with the specific localization of the metastasis, potentially still justifying CDKi use in the case of hepatic involvement. This assumption is highlighted by the results of another RWE study showing that treatment with CDKi led to a significantly better survival compared to chemotherapy despite visceral crisis [44]. 

Another impactful variable was the development of AEs which had no significant association with PFS but with TTF, short-term, and prolonged therapy benefit. Early adverse events ≤ 4 months in particular, negatively impacted the short-term benefit, likely leading to earlier therapy discontinuation. Compared to endocrine therapy alone, the additional use of CDKi results in significantly higher rates of AE, most frequently cytopenia, gastrointestinal complaints, fatigue, and increase in transaminases [45]. In the present study, 15 patients experienced treatment failure without ever developing disease progression under CDKi with the frequent reasons leading to treatment discontinuation being gastrointestinal complaints (n = 6, such as nausea, vomiting, and diarrhea) followed by neutropenia (n = 2) and upon patient request (n = 2). Despite this, therapeutic adjustments such as dose reduction or pausing were only performed in 5 out of these 15 cases (3/6 with gastrointestinal complaints, 1 each with exanthema, and upon patient request) compared to 35 out of the remaining 71 cases who had ongoing treatment or progressive disease (33% vs. 49%, respectively). None out of the 15 cases received a substance switch to another CDKi. These numbers were also reflected in the predictive analyses. Treatment strategies like therapy pause were significant for achieving early benefit, possibly by mitigating early AEs, whereas switching the CDKi was notably effective for securing prolonged benefits beyond 10 months, possibly by achieving prolonged tumor response due to differences in drug efficacy [46]. These insights suggest that managing AEs proactively and strategically adapting treatment approaches in the face of side effects and progression can potentially extend therapy duration and improve long-term outcome.

Lastly, our exploratory analyses identified that the presence of diabetes as comorbidity was significantly associated with poorer TTF. In our cohort, all 11 patients with known diabetes experienced disease progression (n = 6) or AEs leading to therapy discontinuation (n = 5). In clinical trials, patients with diabetes are generally at risk of having higher toxicity to chemotherapy, hospitalization rate, and all-cause mortality [47]. Although no previous clinical trials on CDKi have focused on the role of diabetes, in vivo studies have shown that genetic CDK4 loss as well as treatment with the CDKi Palbociclib lead to the reduction in beta-islet pancreatic cells. This may cause or aggravate insulin-deficient diabetes and thus possibly lead to increased toxicity to CDKi [48,49]. Promising studies on the anti-diabetic drug Metformin indicate the improvement of chemotherapy toxicity and potential heightened efficacy of CDKi in other cancer entities, which should encourage further evaluation in breast-cancer specific studies [50,51].

### 4.4. Implications for Future Research and Clinical Practice

Addressing the study’s limitations, the follow-up duration was too short to capture long-term outcomes and the sample size was relatively small, restricting the study’s power. Additionally, adjuvant antihormonal therapy (AHT) stratification was missing, which could have provided more nuanced insights into treatment efficacy. While using alternative outcome parameters might be seen as a limitation, with our patient-centered perspective, these were essential to reflect the real-world anxiety of therapy changes, justifying their inclusion in our study. The predominance of Palbociclib (86.1%) usage could suggest a lack of balance in treatment representation; however, this is mainly due to the fact that Palbociclib was the first approved CDKi for BC treatment and the present study was focused on the initial phase of CDKi introduction.

The strengths of our study lie in the detailed examination of numerous variables, offering a granular view of RWD. This approach not only mirrors the actual clinical environment, but also unveils practical challenges in patient management. The inclusion of diverse outcome parameters, coupled with an individualized approach based on patient characteristics, allowed for a nuanced understanding of patient needs. It also facilitates personalized advice that incorporates factors beyond disease progression, such as AE management. Our study aligns problem identification, research philosophy, and methodology seamlessly. Observing the varied individual responses to CDKi therapy, we embarked on a study grounded in inductive reasoning, inching closer to understanding why some patients benefit significantly while others do not. 

With regard to future implications, the indication for CDKi should be cautiously considered for previously treated patients, where the advantage over exclusive endocrine therapy remains questionable. The limited benefit for endocrine-resistant individuals calls for larger patient cohorts to confirm findings. Moreover, comparative studies between chemotherapy and CDKi are essential. The predictive significance of AE and therapeutic adjustments by pausing or switching the CDKi prompts a re-evaluation of management strategies. This emphasizes the need for intense therapy support akin to clinical trial conditions, enhancing adherence and addressing therapy-related challenges. Our approach serves as a pilot for more personalized therapy decisions and management, advocating for intensified care in the case of adverse events and potentially switching treatments. This patient-centric strategy, along with better management and adherence practices, warrants further research and implementation. The decision to use CDKi, particularly for patients with low PR levels, diabetes, or at advanced treatment lines, needs careful consideration, informed by real-world data and a deeper understanding of disease biology. In conclusion, as CDKi are explored in various contexts like HER2-positive situations [52], neoadjuvant settings [53], or as maintenance therapy [54], our findings should be integrated into the broader discourse, contributing to a more effective and nuanced application of these therapies in clinical practice [55]. In hindsight of the current results of this study, the next step for further research would be an RCT analyzing the effect of an intensified patient management program mimicking RCT conditions vs. usual care.

## 5. Conclusions

In this single-center RWE study from November 2016 to May 2020, 86 patients were assessed, of which 46.5% (40/86) discontinued treatment due to disease progression and 17.4% (15/86) primarily due to AEs. Our multivariate analysis unveiled significant predictors for PFS and TTF, with a notable median PFS of 12 months and TTF of 8.5 months. High progesterone receptor (PR) expression was positively correlated with extended PFS and TTF, indicating a potential biomarker for treatment response. Multiple metastases and hepatic involvement were identified as adverse factors for PFS, reflecting the complex nature of metastatic BC and its impact on treatment outcomes. Notably, strategic therapy adjustments, such as pauses and switches between CDKi, were associated with improved therapeutic benefits, underscoring the importance of individualized treatment strategies. The study highlighted the role of tumor biology, with high PR levels linked to better outcomes, and low tumor grading emerging as a significant factor for long-term benefit. Early treatment discontinuation due to adverse events and the presence of comorbidities like diabetes were critical considerations, affecting treatment duration and efficacy. In summary, insights gained from our study contributed to bridging the gap between RCT-assessed drug efficacy and clinical reality, advocating for tailored approaches to optimize patient outcomes and highlighting directions for future research.

## Figures and Tables

**Table 1:** Distribution of continuous characteristics of patient cohort.

Variable	Median
Age at metastatic disease (years)	58 (28–86)
Age at start of CDK4/6 inhibitor (years)	66 (33–90)
ER at any time (%)	95 (0–100)
PR at any time (%)	62.5 (0–95)
Ki67 at any time (%)	30 (3–80)
Time between metastasis until CDK4/6 therapy (months)	5 (0–155)
Number of metastatic sites	2 (0–5)
PFS (months)	12 (1–39)
TTF (months)	8.5 (1–39)

**Table 2:** Distribution of categorical baseline characteristics of patient cohort.

Variable	Total = 86 n (%)
**Therapeutic Status**	
Ongoing	28 (32.6)
Failure due to progression	40 (46.5%)
Failure due to AE	15 (17.4%)
Failure due to both	3 (3.5%)
**Treatment Benefit**	
≥4 months	57 (66.3%)
≥10 months	42 (48.8%)
**Occurence of AE**	
At any time	49 (57.0%)
Early (<4 months)	35 (40.7%)
**CDKi therapy**	
First line	39 (45.4%)
Further lines	47 (54.7%)
CDKi substance *	
Palbociclib	74 (86.1%)
Ribociclib	13 (15.1%)
Abemaciclib	5 (5.8%)
**Pausing or switching of CDKi**	
Pause	32 (37.2%)
Switch	5 (5.8%)
**Previous diagnosis of primary breast cancer**	
Yes	57 (66.3%)
No	29 (33.7%)
**Recurrence at the time or before metastatic disease**	
No primary breast cancer	29 (33.7%)
Yes	15 (17.4%)
No	42 (48.8%)
**Grading at any time**	
1	1 (1.2%)
2	56 (65.1%)
3	27 (31.4%)
missing	2 (2.3%)
**Previous treatment at any time**	
Operation	61 (70.9%)
Mastectomy	33 (38.4%)
Axillary Dissection	39 (45.4%)
Radiation	71 (82.6%)
Chemotherapy	59 (68.6%)
Antihormonal therapy	47 (54.7%)
**Metastatic Sites**	
1	30 (34.9%)
2	26 (30.2%)
3	18 (20.9%)
≥4	12 (14.0%)
**Localization of metastasis**	
Bone	71 (82.6%)
Pulmonal/pleural	34 (39.5%)
Hepatic	27 (31.4%)
Nodal	25 (29.1%)
Skin	7 (8.1%)
Brain	6 (7.0%)
Peritoneal	3 (3.5%)
Other	10 (11.6%)
Bone-only	21 (24.4%)
Visceral	52 (60.5%)
**Relevant Comorbidities**	56 (65.1%)
Cardiovascular Diseases	45 (52.3%)
- Arterial hypertension	33 (38.4%)
- Ischemia (incl. myocardial infarction and stroke)	12 (14.0%)
- Venous thrombosis (incl. pulmonary embolism)	11 (12.8%)
- Atrial fibrillation	7 (8.1%)
Non-cardiovascular diseases	18 (20.9%)
- Diabetes	11 (12.8%)
- Previous cancer (excluding breast cancer)	4 (4.7%)

* The total number of CDKi substance is 92 instead of 86, because five patients switched from Palbociclib to Ribociclib and/or Abemaciclib.

**Table 3:** Multivariate analyses for PFS, TTF, treatment benefit ≥ 4 and ≥10 months (total n = 86).

Variable	N (%)	Median CDKi Duration (Months)/ Rate of Treatment Benefit	Hazard/Odds Ratio	95% Confidence Interval	*p*-Value
**PFS**					
**PR**	per 10% increase		HR: 0.880	0.978–0.996	0.006
**Metastatic sites**					
Multiple	30 (34.9%)	5.5 (1–30)	HR: 2.557	1.135–5.763	0.024
Single	50 (65.1%)	10 (1–39)			
**Presence of hepatic metastasis**					
Yes	27 (31.4%)	4 (1–39)	HR: 2.009	1.034–3.903	0.040
No	59 (68.6%)	8.5 (1–20)			
**TTF**					
**PR**	per 10% increase		HR: 0.858	0.792–0.929	0.0002
**Metastatic sites**					
Multiple	30 (34.9%)	5.5 (1–30)	HR: 3.290	1.699–6.369	0.0004
Single	50 (65.1%)	10 (1–39)			
**Occurrence of UAE at any time**					
Yes	49 (57.0%)	6 (1–25)	HR: 2.346	1.318–4.176	0.0037
No	37 (43.0%)	10 (2–39)			
**Diabetes**					
Yes	11 (12.8%)	3 (1–39)	HR: 2.882	1.346–6.171	0.007
No/unclear	75 (87.2%)	7 (1–20)			
**Treatment Benefit ≥ 4 months**					
**PR**	per 10% increase		OR: 1.220	1.047–1.423	0.011
**Occurrence of early AE**					
Yes	35 (40.7%)	35.1% (20/35)	OR: 0.213	0.063–0.716	0.012
No	51 (59.3%)	64.9% (37/51)			
**Pausing of CDKi**					
Yes	32 (37.2%)	81.5% (26/32)	OR: 6.725	1.742–25.963	0.006
No	54 (62.8%)	57.4% (31/54)			
**Time between metastasis until CDKi therapy**					
<median (5 m)	41 (47.7%)	78.1% (32/41)	OR: 3.485	1.150–10.564	0.027
≥median (5 m)	45 (52.3%)	55.6% (25/45)			
**Treatment Benefit ≥ 10 months**					
**Grading**	per increase of 1 grade		OR: 0.155	0.045–0.534	0.003
**Metastatic sites**					
Multiple	30 (34.9%)	37.5% (21/30)	OR: 0.237	0.077–0.723	0.011
Single	50 (65.1%)	70.0% (21/50)			
**Occurrence of AE at any time**					
Yes	49 (57.0%)	36.7% (18/49)	OR: 0.284	0.101–0.794	0.017
No	37 (43.0%)	64.9% (24/37)			
**CDKi switch**					
Yes	5 (5.8%)	80.0% (4/5)	OR: 14.267	1.089–186.96	0.043
No	81 (94.2%)	46.9% (38/81)			

**Table 4:** Differences of the patient cohort between randomized controlled trials and the present RWE study.

Study	PALOMA-2	MONALEESA-2	MONARCH-3	Present RWE Study
ECOG	0–1	0–1	0–2	No specified limitation
postmenopausal	100%	100%	100%	80%
first-line CDKi	100%	100%	100%	45%
endocrine backbone	Letrozol	Letrozol	Letrozol	Letrozol or Fulvestrant
prior neoadjuvant or adjuvant chemotherapy	48%	49%	38%	69%
prior chemotherapy in metastatic disease	none	none	none	36%
metastatic spread	Visceral: 48% Bone-only: 23%	Liver or lung: 54% Bone-only: 21%	Visceral: 53% Bone-only: 21%	Visceral: 61% Bone-only: 24%
dose reduction	67%	58%	47%	23%

## Data Availability

The data presented in this study are available on request from the corresponding author. The data are not publicly available due to privacy.

## Supplementary Materials

The following supporting information can be downloaded at: https://www.mdpi.com/article/10.3390/cancers16091760/s1, File S1: List of variables included in the multivariate model building; Table S1: Grouping analyses for PFS, TTF, treatment benefit ≥ 4 and ≥10 months depending on tumor biology, metastatic sites, and previous treatments (total n = 86).

## Abbreviations

BC: breast cancer; CDK: cyclin-dependent kinase; CDKi: cyclin-dependent kinase in-hibitor/inhibitors; CI: confidence interval; PFS: progression-free survival; TTF: time-to-treatment failure; HR: hazard ratio; ET: endocrine therapy, ER: estrogen receptor; DM: diabetes mellitus; PR: progesterone receptor; FDA: US Food and Drug Administration, RCT: randomized controlled trial; RWD: real-world data, RWE: real-world evidence; ECOG: Eastern Cooperative Oncology Group; OR: odds ratio; AE: adverse event.
