# Supplementary material for: Real-World Data Analysis of CDK4/6 Inhibitor Therapy—A Patient-Centric Single Center Study
Source: Cancers (Basel). 2024 May 1;16(9):1760. doi: 10.3390/cancers16091760 (PMC11083990; doi:10.3390/cancers16091760)
Supplement: Supplementary file 1 [file cancers-16-01760-s001.zip › cancers-2965205-File S1.pdf]

File S1:

List of variables included in the multivariate model building

Continuous variables:

1. Age at first diagnosis of breast cancer (per 1 year increase)
2. Age at start of CDK4/6 inhibitor (per 1 year increase)
3. Age at diagnosis of metastasis (per 1 year increase)
4. Time between diagnosis of metastasis until start of CDK4/6-inbibitor (per 1 month increase)
5. Time between first diagnosis of breast cancer until diagnosis of metastasis (per 1 month increase)
6. Time between first diagnosis of breast cancer until diagnosis of metastasis (per 1 year increase)
7. Ki67 (per 1 and 10% increase)
8. Estrogen receptor (per 1 and 10% increase)
9. Progesterone receptor (per 1 and 10% increase)
10. Grading (per 1 grade increase)
11. Number of metastatic sites (per 1 site increase)
12. Time of previous antihormonal therapy (per 1 month increase)
13. Line of therapy when CDK4/6 was initiated (per 1 line increase)
14. BMI (per 1 increase)

Categorical Variables:

Two Categories

1. Age at first diagnosis of breast cancer < 58 vs. ≥ 58 years (median)
2. Age at start of CDK4/6 inhibitor < 66 vs. ≥ 66 years (median)
3. Age at diagnosis of metastasis < 64 vs. ≥ 64 years (median)
4. Time between diagnosis of metastasis until start of CDK4/6-inbibitor < 5 vs. ≥ 5 months
5. Time between first diagnosis of breast cancer until diagnosis of metastasis < 55.5 vs. ≥ 55.5 months
6. Time between first diagnosis of breast cancer until diagnosis of metastasis < 4 vs. ≥ 4 years
7. Ki67 < 30% vs. ≥ 30%
8. Estrogen receptor status < 95% vs. ≥ 95%
9. Progesterone receptor status < 62.5% vs. ≥ 62.5%
10. Grading: G1/G2 vs. G3
11. Time of previous antihormonal therapy < 48 vs. ≥ 48 months
12. BMI < 26.93 vs ≥ 26.93 (median)
13. BMI < 25 vs ≥ 25
14. CDK4/6-inhibitor as first-line vs. further-line therapy
15. Adverse events: yes vs. no
16. Early adverse events: yes vs. no
17. Dose reduction: yes vs. no
18. Pausing of CDK4/6-inhibitor during any time of the treatment: yes vs. no
19. Switching of CDK4/6-inhibitor to another CDK4/6-inhibitor: yes vs. no
20. Metastasis at first diagnosis (primary) vs. Metastasis after previous diagnosis of localized breast cancer (secondary)
21. Switch of receptor status in metastatic disease: yes vs. no
22. Multiple metastatic sites vs. single metastatic site
23. Bone metastasis: yes vs. no
24. Nodal metastasis: yes vs. no
25. Hepatic metastasis: yes vs. no
26. Pulmonary metastasis: yes vs. no
27. Pleural metastasis: yes vs. no
28. Peritoneal metastasis: yes vs. no
29. Skin metastasis: yes vs. no

30. Brain metastasis: yes vs. no
31. Other location of metastasis: yes vs. no
32. Pulmonary or pleura metastases: yes vs. no
33. Visceral metastases: yes vs. no
34. Bone-only disease: yes vs. no
35. Peri-/Postmenopausal vs. premenopausal at primary diagnosis of localized breast cancer
36. Peri-/Postmenopausal vs. premenopausal at diagnosis of metastasis
37. Locoregional Recurrence at time of metastasis: yes vs. no
38. Operation at any time of the disease: yes vs. no
39. Mastectomy at any time of the disease: yes vs. no
40. Axillary dissection at any time of the disease: yes vs. no
41. Radiation at any time of the disease: yes vs. no
42. Radiation of metastases: yes vs. no
43. Radiation of metastases before CDK4/6-inhibitor: yes vs. no
44. Radiation of metastases during CDK4/6-inhibitor: yes vs. no
45. Endocrine resistance: yes vs. no
46. Positive family history: yes vs. no
47. Relevant comorbidities: yes vs. no
48. Smoking: yes vs. no
49. Diabetes mellitus: yes vs. no
50. Other oncological diagnoses: yes vs. no

Three Categories:

1. Ductal carcinoma in situ during first diagnosis of localized breast cancer: yes vs. no vs. primary metastasis
2. Locoregional Recurrence before metastasis: yes vs. no vs. primary metastasis
3. Mastectomy for the initial treatment of localized breast cancer: yes vs. no vs. primary metastasis
4. Axillary dissection for the initial treatment of localized breast cancer: yes vs. no vs. primary metastasis
5. Radiatio for the initial treatment of localized breast cancer: yes vs. no vs. primary metastasis
6. Chemotherapy for the initial treatment of localized breast cancer: yes vs. no vs. primary metastasis
7. Neoadjuvant chemotherapy at diagnosis of localized breast cancer: yes vs. no vs. primary metastasis
8. Antihormonal therapy for the initial treatment of localized breast cancer: yes vs. no vs. primary metastasis
9. Locoregional Recurrence before/at time of metastasis: yes vs. no vs. primary metastasis
10. Type of antihormonal therapy used for the initial treatment of localized breast cancer: Aromatase inhibitor only vs. Tamoxifen only vs. both
