# Supplementary material for: Real-World Data Analysis of CDK4/6 Inhibitor Therapy—A Patient-Centric Single Center Study
Source: Cancers (Basel). 2024 May 1;16(9):1760. doi: 10.3390/cancers16091760 (PMC11083990; doi:10.3390/cancers16091760)
Supplement: Supplementary file 1 [file cancers-16-01760-s001.zip › cancers-2965205-Table S1.pdf]

Table S1. Grouping Analyses for PFS, TTF, Treatment benefit ≥ 4 and ≥ 10 months depending on tumorbiology, metastatic sites and previous treatments (total n = 86).

Variable	N (%)	PFS	TTF	Treatment Benefit ≥ 4 months	Treatment Benefit ≥ 10 months
**Tumorbiology**					
ER per 10% increase	82 (95.3%)	HR: 0.852 (0.752 – 0.966) p = 0.012	HR: 0.851 (0.767 – 0.944) p = 0.002	OR: 1.180 (0.963 – 1.447) p = 0.110	OR: 1.351 (1.044 – 1.750) p = 0.022
ER per 1% increase	82 (95.3%)	HR: 0.984 (0.972 – 0.997) p = 0.012	HR: 0.984 (0.974 – 0.994) p = 0.002	OR: 1.017 (0.996 – 1.038) p = 0.110	OR: 1.031 (1.004 – 1.058) p = 0.022
PR per 10% increase	82 (95.3%)	HR: 0.875 (0.801 – 0.956) p = 0.003	HR: 0.885 (0.821 – 0.954) p = 0.001	OR: 1.156 (1.016 – 1.315) p = 0.028	OR: 1.151 (1.020 – 1.305) p = 0.023
PR per 1% increase	82 (95.3%)	HR: 0.987 (0.978 – 0.995) p = 0.003	HR: 0.988 (0.980 – 0.995) p = 0.001	OR: 1.015 (1.002 – 1.028) p = 0.028	OR: 1.014 (1.002 – 1.027) p = 0.023
Ki-67 per 10% increase	70 (81.4%)	HR: 1.067 (0.881 – 1.293) p = 0.504	HR: 1.129 (0.964 – 1.321) p = 0.132	OR: 0.913 (0.700 – 1.191) p = 0.504	OR: 0.804 (0.615 – 1.053) p = 0.113
Ki-67 per 1% increase	70 (81.4%)	HR: 1.007 (0.987 – 1.026) p = 0.504	HR: 1.012 (0.996 – 1.028) p = 0.132	OR: 0.991 (0.965 – 1.018) p = 0.504	OR: 0.978 (0.953 – 1.005) p = 0.113
**Metastatic site**					
Bone	71 (82.6%)	HR: 1.339 (0.590 – 3.037) p = 0.485	HR: 1.563 (0.736 – 3.319) p = 0.245	OR: 0.669 (0.193 – 2.321) p = 0.527	OR: 0.579 (0.186 – 1.798) p = 0.345
Pulmonal/pleural	34 (39.5%)	HR: 1.617 (0.887 – 2.950) p = 0.117	HR: 1.380 (0 819 – 2.324) p = 0 226	OR: 0.890 (0.358 – 2.215) p = 0.803	OR: 0.731 (0.307 – 1.742) p = 0.480
Hepatic	27 (31.4%)	HR: 3.175 (1.726 – 5.840) p = 0.0002	HR: 2.379 (1.399 – 4.043) p = 0.001	OR: 0.317 (0.122 – 0.823) p = 0.018	OR: 0.240 (0.088 – 0.656) p = 0.005
Nodal	25 (29.1%)	HR: 2.647 (1.421 – 4.929) p = 0.002	HR: 2.057 (1.201 – 3.522) p = 0.009	OR: 0.416 (0.160 – 1.097) p = 0.077	OR: 0.289 (0.105 – 0.793) p = 0.016
Skin	7 (8.1%)	HR: 0.983 (0.302 – 3.196) p = 0.977	HR: 0.988 (0.356 – 2.739) p = 0 981	OR: 3.294 (0.377 – 28.747) p = 0.281	OR: 0.769 (0.162 – 3.663) p = 0.742
Brain	6 (7.0%)	HR: 1.031 (0.318 – 3.348) p = 0.959	HR: 1.024 (0.370 – 2.836) p = 0.964	OR: 1.019 (0.175 – 5.919) p = 0.983	OR: 0.500 (0.087 – 2.886) p = 0.438
Peritoneal	3 (3.5%)	HR: 0.997 (0.240 – 4.138) p = 0.997	HR: 1.147 (0.358 – 3.678) p = 0.818	OR: > 999.9 (<0.01– > 999.9) p = 0.978	OR: 2.150 (0.188 – 24.633) p = 0.539
Other	10 (11.6%)	HR: 0.803 (0.337 – 1.913) p = 0.620	HR: 0.715 (0.323 – 1.583) p = 0.408	OR: 5.250 (0.631 – 43.647) p = 0.125	OR: 1.667 (0.435 – 6.383) p = 0.4556
Bone-only	21 (24.4%)	HR: 0.331 (0.139 – 0.788)	HR: 0.343 (0.162 – 0.725)	OR: 3.999 (1.069 – 14.958)	OR: 3.519 (1.210 – 10.233)

		p = 0.012	p = 0.005	p = 0.039	p = 0.021
Visceral	52<br>(60.5%)	HR: 2.246<br>(1.166 – 4.325)<br>p = 0.016	HR: 2.229<br>(1.261 – 3.938)<br>p = 0.006	OR: 0.454<br>(0.173 – 1.194)<br>p = 0.110	OR: 0.419<br>(0.173 – 1.017)<br>p = 0.055
**Previous treatment at any time**					
Operation	61<br>(70.9%)	HR: 1.860<br>(0.910 – 3.802)<br>p = 0.089	HR: 2.079<br>(1.095 – 3.948)<br>p = 0.025	OR: 0.522<br>(0.182 – 1.497)<br>p = 0.226	OR: 0.418<br>(0.160 – 1.093)<br>p = 0.075
Mastectomy	33<br>(38.4%)	HR: 1.742<br>(0.947 – 3.205)<br>p = 0.074	HR: 1.764<br>(1.045 – 2.978)<br>p = 0.034	OR: 0.345<br>(0.137 – 0.872)<br>p = 0.024	OR: 0.438<br>(0.179 – 1.070)<br>p = 0.070
Axillary Dissection	39<br>(45.4%)	HR: 1.387<br>(0.760 – 2.532)<br>p = 0.287	HR: 1.233<br>(0.734 – 2.072)<br>p = 0.428	OR: 0.679<br>(0.276 – 1.667)<br>p = 0.398	OR: 0.822<br>(0.351 – 1.923)<br>p = 0.651
Chemotherapy	71<br>(82.6%)	HR: 2.151<br>(1.027 – 4.502)<br>p = 0.042	HR: 1.906<br>(1.025 – 3.544)<br>p = 0.042	OR: 0.762<br>(0.285 – 2.036)<br>p = 0.588	OR: 0.433<br>(0.170 – 1.103)<br>p = 0.079
Radiotherapy	59<br>(68.6%)	HR: 3.642<br>(1.118– 11.869)<br>p = 0.032	HR: 2.752<br>(1.093 – 6.930)<br>p = 0.032	OR: 0.669<br>(0.193 – 2.321)<br>p = 0.527	OR: 0.410<br>(0.127 – 1.323)<br>p = 0.136
Antihormonal Therapy during primary disease	47<br>(54.7%)	HR: 1.586<br>(0.555 – 4.531)<br>p = 0.389	HR: 1.012<br>(0.469 – 2.180)<br>p = 0.976	OR: 1.176<br>(0.291 – 4.762)<br>p = 0.382	OR: 1.884<br>(0.433 – 8.190)<br>p = 0.941
Tam<br>(vs. AI upfront)	18<br>(21.4%)	HR: 1.547<br>(0 585 – 4.091)<br>p = 0.379	HR: 1.292<br>(0.566 – 2.950)<br>p = 0.543	OR: 0.786<br>(0.188 – 3.290)<br>p = 0.741	OR: 0.533<br>(0.133 – 2.141)<br>p = 0.375
Tam/AI-combination<br>(vs. AI upfront)	12<br>(14.3%)	HR: 2.842<br>(1.000 – 8.075)<br>p = 0.050	HR: 2.188<br>(0.896 – 5.346)<br>p = 0.086	OR: 0.700<br>(0.145 – 3.370)<br>p = 0.656	OR: 0.533<br>(0.133 – 2.141)<br>p = 0.032

Abbrevations: ER: estrogen receptor, PR: progesterone receptor, AI: aromatase inhibitor
